# Hypotension Prediction Index guided Goal Directed therapy and the amount of Hypotension during Major Gynaecologic Oncologic Surgery: a Randomized Controlled clinical Trial

**DOI:** 10.1007/s10877-023-01017-1

**Published:** 2023-04-29

**Authors:** Luciano Frassanito, Pietro Paolo Giuri, Francesco Vassalli, Alessandra Piersanti, Manuel Ignacio Monge Garcia, Chiara Sonnino, Bruno Antonio Zanfini, Stefano Catarci, Massimo Antonelli, Gaetano Draisci

**Affiliations:** 1 Department of Scienze Dell’Emergenza, Anestesiologiche e Della Rianimazione, IRCCS Fondazione Policlinico A. Gemelli, Rome, Italy; 2 Department of Critical Care and Perinatal Medicine, Istituto Di Ricovero e Cura a Carattere Scientifico (IRCCS) Istituto Giannina Gaslini, Genova, Italy; 3 Critical Care Unit, Hospital Universitario de Jerez de La Frontera, Jerez de La Frontera, Cádiz, Spain

**Keywords:** General Anesthesia, Hypotension/prevention & control, Intraoperative Complications/diagnosis, Machine Learning, Gynecologic Neoplasm

## Abstract

Intraoperative hypotension (IOH) is associated with increased morbidity and mortality. Hypotension Prediction Index (HPI) is a machine learning derived algorithm that predicts IOH shortly before it occurs. We tested the hypothesis that the application of the HPI in combination with a pre-defined Goal Directed Therapy (GDT) hemodynamic protocol reduces IOH during major gynaecologic oncologic surgery. We enrolled women scheduled for major gynaecologic oncologic surgery under general anesthesia with invasive arterial pressure monitoring. Patients were randomized to a GDT protocol aimed at optimizing stroke volume index (SVI) or hemodynamic management based on HPI guidance in addition to GDT. The primary outcome was the amount of IOH, defined as the timeweighted average (TWA) mean arterial pressure (MAP) < 65 mmHg. Secondary outcome was the TWA-MAP < 65 mmHg during the first 20 min after induction of GA. After exclusion of 10 patients the final analysis included 60 patients (30 in each group). The median (25–75th IQR) TWA-MAP < 65 mmHg was 0.14 (0.04–0.66) mmHg in HPI group versus 0.77 (0.36–1.30) mmHg in Control group, P < 0.001. During the first 20 min after induction of GA, the median TWA-MAP < 65 mmHg was 0.53 (0.06–1.8) mmHg in the HPI group and 2.15 (0.65–4.2) mmHg in the Control group, P = 0.001. Compared to a GDT protocol aimed to SVI optimization, a machine learning-derived algorithm for prediction of IOH combined with a GDT hemodynamic protocol, reduced IOH and hypotension after induction of general anesthesia in patients undergoing major gynaecologic oncologic surgery.

Trial registration number: NCT04547491. Date of registration: 10/09/2020.

## Introduction

Intraoperative hypotension (IOH) represents a common event during noncardiac surgery and has been associated with worse postoperative outcomes [1–3]. Several studies showed a significant relationship between IOH and increased incidence of postoperative acute kidney injury, myocardial injury, and 30-days mortality [4–9]. Organ injuries and postoperative complications appear to be related to the depth, frequency, and duration of hypotensive episodes [8, 10, 11]. Specifically, the most recent literature showed a strong association between IOH (defined as mean arterial pressure—MAP—lower than 65 mmHg) and higher risk of morbidity [5, 8, 10–12]. A large number of patients undergoing major surgery experience at least one episode of IOH, and about a third of these events occurs between induction of general anesthesia (GA) and surgical incision [13–15].

The use of cardiac output (CO) monitoring to guide administration of intravenous fluid and inotropic drugs as part of a hemodynamic therapy algorithm has been shown to improve tissue perfusion and oxygenation [16]. The so-called Goal Directed haemodynamic Therapy (GDT) refers to the use of a protocol to optimise CO-based haemodynamic targets and to the treatments used to reach these targets [17].

The multicentric INPRESS randomized controlled trial (RCT) demonstrated that applying an individualized blood pressure target to be achieved with norepinephrine infusion in addition to a GDT reduces postoperative organ dysfunction, suggesting that using a target approach to minimize IOH would reduce organ injury [18].

Despite careful hemodynamic monitoring and protocols for vasopressor and fluid administration, accurate control of MAP remains a challenge [10, 18, 19]. According to several authors fluid optimization before induction of GA and during elective surgery has a minor impact on hypotensive events occurring immediately after induction and during surgery [13, 15, 20].

It is difficult to predict IOH. Current treatment is often initiated when hypotension is already manifest, and anaesthesiologists only react to it. An algorithm developed using machine learning techniques and based on the arterial pressure waveform analysis, named Hypotension Prediction Index (HPI, Edwards Lifesciences, Irvine, USA), has been recently developed [21]. The HPI algorithm provides the anaesthesiologist a unitless number from 0 to 100 that increases accordingly to the risk of developing a hypotensive event in the near future. HPI has been validated on surgical patients with a high sensitivity and specificity in predicting hypotension 5, 10 and 15 min before the event [22]. Authors who tried to reduce IOH through an HPI-based management protocol reported mixed results [23–25] and a recent metanalysis revealed that only low quality evidence is available to determine the benefit of such management [26].

Major gynaecologic oncologic surgery for cancer mass reduction is often associated with unstable hemodynamics and significant blood loss [27, 28]. Hypotension during gynaecologic oncologic surgery is common and is associated with the potential for harm. In a previous pilot study, we reported that 77.4% of patients undergoing gynaecologic oncologic surgery experienced at least 1 hypotensive event [29] and that the application of a HPI-based hemodynamic management protocol was associated with reduced hypotensive burden compared with standard care [30].

The primary aim of this single-centre RCT was to compare the cumulative amount of IOH (defined as a MAP value below 65 mmHg) in 2 groups of patients undergoing major gynaecologic oncologic surgery managed using a GDT protocol aimed to optimize cardiac output or the HPI hemodynamic guidance combined to a different GDT protocol. Secondly, we compared IOH during the first 20 min after induction of GA and the amount of severe hypotension in the 2 groups. In addition, we also tested the threshold of 50 mmHg for IOH.

## Materials and methods

This study was a single-centre RCT registered at ClinicalTrials.gov (identifier NCT04547491) and conducted at the IRCCS Policlinico Universitario Agostino Gemelli Foundation (Rome, Italy) in accordance with Good Clinical Practice guidelines and the principles of the Declaration of Helsinki. The study was approved by the Institutional Ethics Committee (ID 3672, protocol N. 0049955/20) and registered in ClinicalTrials.gov (identifier NCT04547491). The full protocol and datasets are available at lucfras75@hotmail.com on a collaborative basis.

The inclusion criteria were patients ≥ 18 years old with American Society of Anesthesiologists (ASA) physical status II-IV, scheduled to major gynaecologic oncologic surgery with expected duration > 2 h under GA and planned continuous invasive blood pressure monitoring. Exclusion criteria were significant cardiac arrhythmias, such as permanent atrial fibrillation, aortic regurgitation, coagulation disorders, emergency surgery, preoperative infection, the requirement of dialysis, contraindication to radial artery cannulation and patient’s refusal of the treatment of personal data.

Eligibility of consecutive patients scheduled to major gynaecologic oncologic surgery fulfilling the inclusion criteria was assessed by an investigator involved in the trial based on preoperative medical records. Written informed consent was obtained from the enrolled patients the day prior to surgery by a study staff member and enrolment ceased when the target sample size was obtained.

Randomization codes to the HPI group or to the GDT group (Control group) were generated, in a 1:1 ratio, by an independent research team member, using a reproducible web-based system that uses the pseudo-random number generator of Wichmann and Hill as modified by McLeod (Randomization.com) and they were stored in sequentially numbered, opaque, sealed and stapled envelopes. The day of surgery, a researcher not involved in clinical care opened the sequentially numbered envelopes and patients were blinded to group allocation.

On arrival at the operating room, a large-bore venous catheter was inserted in a forearm vein. Standard monitoring (Life Scope TR, Nihon Kohden Co, Tokyo, Japan) included a 5-lead electrocardiogram, pulse oximetry and a non-invasive blood pressure cuff placed on the left arm. In all study participants, after mild sedation with midazolam and local infiltration with Lidocaine 2%, an arterial catheter was placed in the right radial artery before induction.

Patients in the HPI group received invasive blood pressure monitoring with the Acumen IQ sensor transducer connected to the HemoSphere platform (Edwards Lifesciences, Irvine, CA). Patients in the Control group had a Flotrac sensor transducer connected to the EV1000 platform (Edwards Lifesciences). In both groups, the arterial pressure waveform was measured continuously with a sampling rate of 100 Hz. The HemoSphere and the EV1000 monitors displayed hemodynamic parameters calculated from the waveform every 20 s, including the HPI (that was detected only by the HemoSphere used in the HPI group). The signal quality of the arterial waveform was carefully checked with a fast flush test when starting the monitoring. Hemodynamic parameters displayed on both dedicated monitors (HemoSphere for HPI-group and EV1000 for Control-group) included mean, systolic and diastolic arterial pressure (MAP, SBP, DBP), heart rate (HR), stroke volume (SV), stroke volume index (SVI), stroke volume variation (SVV), pulse pressure variation (PPV), CO and cardiac index (CI).

All patients were offered central neuraxial anesthesia for postoperative pain management, either intrathecal morphine 100 μg for expected laparoscopic surgery or T12-L1 epidural catheter placement for expected laparotomic surgery.

Induction of GA was performed with propofol 2–3 mg·kg^−1^, sufentanil 0.2 mcg·kg^−1^ and rocuronium bromide 0.6 mg·kg^−1^. Sevoflurane was used for maintenance, to achieve a target bispectral index (BIS) value between 40 and 50. Intravenous sufentanil between 0.1 and 0.2 mcg·kg^−1^·hr^−1^ was supplemented. Mechanical ventilation was performed with a tidal volume of 8 ml ·kg^−1^ of predicted body weight, with a positive end-expiratory pressure of 5 cmH2O, and an inspired oxygen fraction to maintain oxygen saturation ≥ 96%. The respiratory rate was adjusted to maintain end-tidal CO_2_ between 35 and 40 mmHg. An infusion of 3 ml·kg^−1^ ·hr^−1^ of Ringer Lactate (RL) solution was started as fluid maintenance.

**Fig. 1:**
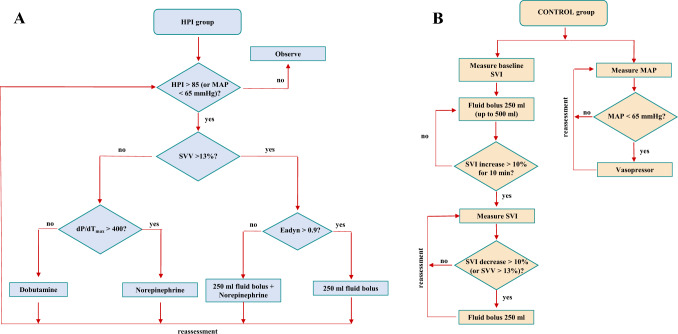
Treatment algorithm for the two groups. In HPI group (Panel **A**) Norepinephrine, when required, was started at dose of ·0.1 mcg·kg-1·min-1 and increased if necessary. Dobutamine was started at dose of 2.5 mcg·kg-1·min-1 and increased if necessary. In the Control group (Panel **B**) after induction of GA, the maximal value of SVI was defined as the absence of a sustained rise in SVI of at least 10% for more than 10 min in response to a fluid challenge (up to 500 ml). Further 250 ml fluid challenges were administered only when SVI decreased > 10% or when SVV was > 13%. At the same time if MAP was < 65 mmHg vasoactive drugs were administered. Abbreviations: HPI: Hypotension Prediction Index. MAP: mean arterial pressure. SVV: stroke volume variation. dP/dT_max_: peak rate of arterial pressure. Ea_dyn_: dynamic arterial elastance SVI: stroke volume index

All physicians involved in the study were trained on the use of the HPI and were informed about the study protocol. In the HPI group, the HPI parameter was displayed on the HemoSphere screen in addition to other hemodynamic parameters. Intraoperatively, a researcher was dedicated to recording any details related to the surgery or anesthesia. When the HPI reached the value of 85 or more, the number blinked red, and an audible alarm alerted the anaesthesiologist to the risk of hypotension. In this eventuality, the HemoSphere monitor with the Acumen software displayed a secondary screen with the following additional variables: SVV (already present in the basic screen), the peak rate of arterial pressure (dP/dt_max_), dynamic arterial elastance (Ea_dyn_, defined as PPV/SVV), providing information about the underlying cause of the impending hemodynamic instability. To standardize the interpretation of hemodynamic parameters, a therapeutical GDT “modified” protocol was established (Fig. 1, Panel A), which considers the main mechanisms of hypotension (hypovolemia, vasoplegia, and decreased contractility). Recommended potential interventions were fluids, fluids plus vasopressor, vasopressor, or inotrope.

In the Control group the anaesthesiologists applied perioperative GDT according to the institutional optimization protocol (Fig. 1, Panel B). After induction of GA, the maximal value of SVI was defined as the absence of a sustained rise in SVI of at least 10% for more than 10 min in response to a fluid challenge. No more than 500 ml of RL was administered for the initial determination of the maximal value of SVI before the beginning of the surgical procedure. Further 250 ml fluid challenges were administered only when SVI decreased > 10% or when SVV was > 13%. SVI optimization was maintained during surgery with subsequent boluses of fluids as required. At the same time if MAP was < 65 mmHg vasoactive drugs were administered. According to routine GDT protocol of our hospital, dobutamine was not scheduled for routine use.

The primary outcome measure was the time-weighted average (TWA) MAP under 65 mmHg during surgery. The TWA combines the number, duration, and severity of hypotensive events, corrected for the total time of measurement (“amount of hypotension”) [29]. Secondary outcomes included the TWA-MAP < 65 mmHg during the first 20 min after induction of GA, and the TWA under the threshold of 50 mmHg (severe hypotension) throughout monitoring time, the total number of hypotensive events per patient, total time with MAP < 65 mmHg, and percentage of time spent with MAP < 65 mmHg with respect to the total duration of the surgery.

In order to evaluate the potential risk of overtreatment in the HPI group, we included the incidence of hypertension (defined as MAP > 110 mmHg) and severe hypertension (MAP > 130 mmHg) as the TWA-MAP above the thresholds of 110 and 130 mmHg during the entire monitoring time [23].

Treatment choice (vasopressor, inotrope, fluids, erythrocyte transfusion) and cumulative administered dose in response to an alarm in the HPI group and to hypotension in the Control group was also evaluated and compared, as well as time from alarm to start of an intervention in the HPI group and from the onset of hypotension to start of treatment in the Control group. An alarm was deemed present when the HPI prediction value reached 85 or higher for at least 1 min and ended when the value normalized (< 85) for at least 1 min. Time to first intervention was used in case of multiple treatments to an alarm or hypotensive event; all alarms or hypotensive events per patient were considered for this analysis. All alarms or hypotensive events per patient were used for the analysis.

Intraoperative exploratory outcomes included the amount of crystalloid and colloid infusion, and cumulative dose of vasoactive drugs. Postoperative exploratory outcomes included the occurrence of major complications before hospital discharge, and mortality at 30 days.

### Sample size calculation

Based on previous results [25], we estimated a mean TWA-MAP < 65 mmHg of 0.5 mmHg in the.control group with 0.51 mmHg of standard deviation. A 75% reduction of mean TWA-MAP < 65 mmHg in the intervention group (i.e. mean 0.12 mmHg) was considered to be a significant effect of the HPI algorithm. An effect size of 0.74 resulted from dividing the mean difference between groups (0.38 mmHg) by the standard deviation. Therefore, a sample size of 60 patients, 30 in each group, would provide 80% power to detect this effect using a 2-group t-test with an α = 0.05 2-sided significance.

### Statistical analysis

Continuous data are presented as medians with interquartile ranges (25th to 75th IQR). Categorical data are presented as frequencies with percentages. Normality distribution of a variable was assessed graphically and with the Shapiro–Wilk test.

Hypotensive events (defined as a MAP < 65 mmHg for > 1 min) and severe hypotensive events (defined as a MAP < 50 mmHg for > 1 min) were analyzed in terms of number, duration, area under the threshold of 65 mmHg and 50 mmHg, and TWA of the area under the threshold. The area under the curve (AUC) MAP below a threshold was calculated as the cumulative sum of the areas below the given threshold for a patient using the trapezoid rule and measured in units of mmHg times minutes [29]. TWA-MAP below the threshold for each patient was derived by dividing AUC-MAP by the time interval between the first and the last MAP measurements (monitoring time). TWA-MAP is expressed in units of mmHg: TWA = (depth of hypotension in mmHg below a MAP threshold × time in minutes spent below the threshold) ÷ total monitoring time in minutes (or 20 min after anesthesia induction) [31]. TWA is similar to a weighted arithmetic mean: two MAP measurements with a longer time interval in-between those contributed more into the TWA than 2 MAP measurements with shorter time interval in between. Calculation of the specific area started when MAP was under the threshold and ended when MAP was higher. The same method was used to quantify hypertension (TWA-MAP above the threshold of 110 and 130 mmHg).

Continuous primary and secondary outcomes were compared using 2-sample Wilcoxon rank-sum test and Hodges Lehman estimation of location shift with corresponding asymptotic 95% CI, as the variables were not normally distributed. Differences on secondary categorical outcomes were assessed with the Chi-square test or Fisher’s exact test in case of expected frequencies < 5.

Postoperative explorative outcomes including major complications before hospital discharge and 30-day mortality were evaluated by reviewing in-hospital electronic medical records or by contacting the patients by telephone and were reported as number and proportion.

Data analysis was performed using R (R Foundation for Statistical computing, Austria, version 4.1.2), Matlab (The MathWorks Inc, Natick, MA, USA) and Acumen Analytics software (Edwards Lifesciences). A 2-sided probability value of *P* < 0.05 was considered statistically significant.

## Results

**Fig. 2:**
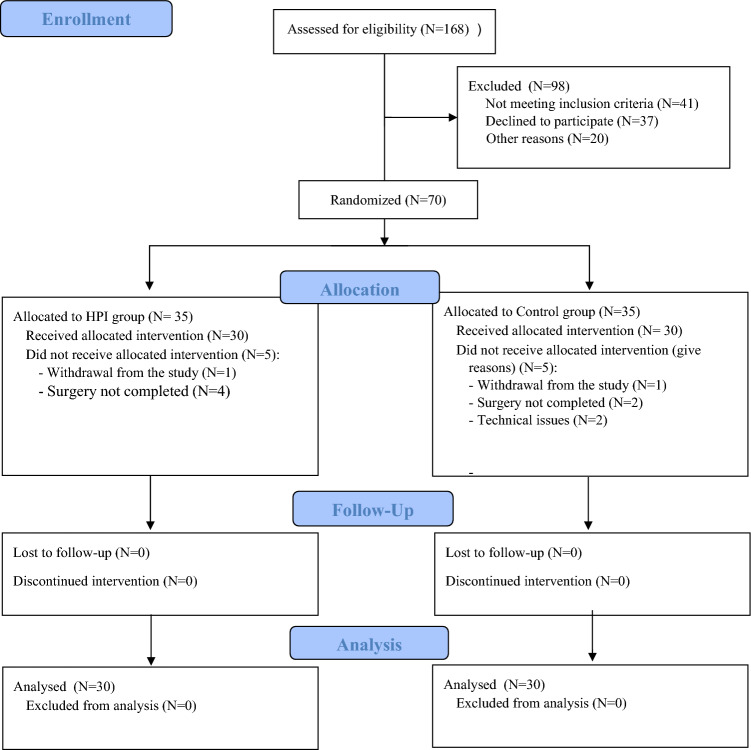
Participants’ Flow Diagram

A total of 70 patients were enrolled between December 2020 and May 2021. Of these, 35 patients were randomized to HPI group and 35 to Control group; 5 patients for each group were excluded from analysis, as shown in Fig. 2. In 6 patients (4 for HPI group, 2 for Control group) surgery was not completed.

The median age was 59 years (48 to 69). Table 1 shows the baseline characteristics of both study groups. The median monitoring time was 253 min (201–324 min) in the HPI group and 325 min (220– 387 min) in the Control group, with a Hodges-Lehman estimator of − 43 min (95% CI: − 110 to 11 min, *P* = 0.147).

**Table 1:** Demographic, Baseline and surgical characteristics of the study population (n = 60)

Baseline characteristics			
Characteristic	HPI group (N = 30)	Control group (N = 30)	Absolute Standardized Difference
Age, y	55 (45, 72)	59 (49, 68)	0.078
Height, m	1.62 (1.58, 1.65)	1.60 (1.55, 1.64)	0.308
Weight, Kg	60 (55, 70)	58 (55, 68)	0.267
Body mass index, Kg/m^2^	23 (21, 26)	22 (20, 25)	0.131
Body surface area, m^2^	1.7 (1.6, 1.7)	1.6 (1.5, 1.7)	0.315
ASA status			0.094
1	0	0	
2	24 (80)	25 (83)	
3	5 (17)	4 (14)	
4	1 (3)	1 (3)	
Medical history			
Cardiovascular diseases	15 (50)	9 (30)	0.417
Pulmonary diseases	7 (23)	6 (20)	0.081
Neurologic diseases	7 (23)	7 (23)	0
Diabetes	1 (3)	2 (6)	0.153
Medical history			
β-blockers	2 (6)	1 (3)	0.153
Angiotensin-converting enzyme inhibitors	6 (20)	5 (17)	0.086
Anticoagulants/Antiplatelet agents	5 (17)	4 (14)	0.093
Diuretics	6 (20)	3 (10)	0.283
Statins	3 (10)	2 (6)	0.121
Oral hypoglicemic agents	1 (3)	2 (6)	0.153
Epidural analgesia	7 (23)	8 (26)	0.077
Subarachnoid analgesia	16 (53)	12 (40)	0.270
Surgical approach			0.706
Laparoscopic	15 (50)	6 (20)	
Laparotomy	5 (17)	5 (17)	
Combined	10 (33)	19 (63)	

Data are presented as N (%) or median (25th, 75th IQR)

*HPI* Hypotension Prediction Index, *ASA* American Society of Anaesthesiologists

**Fig. 3:**
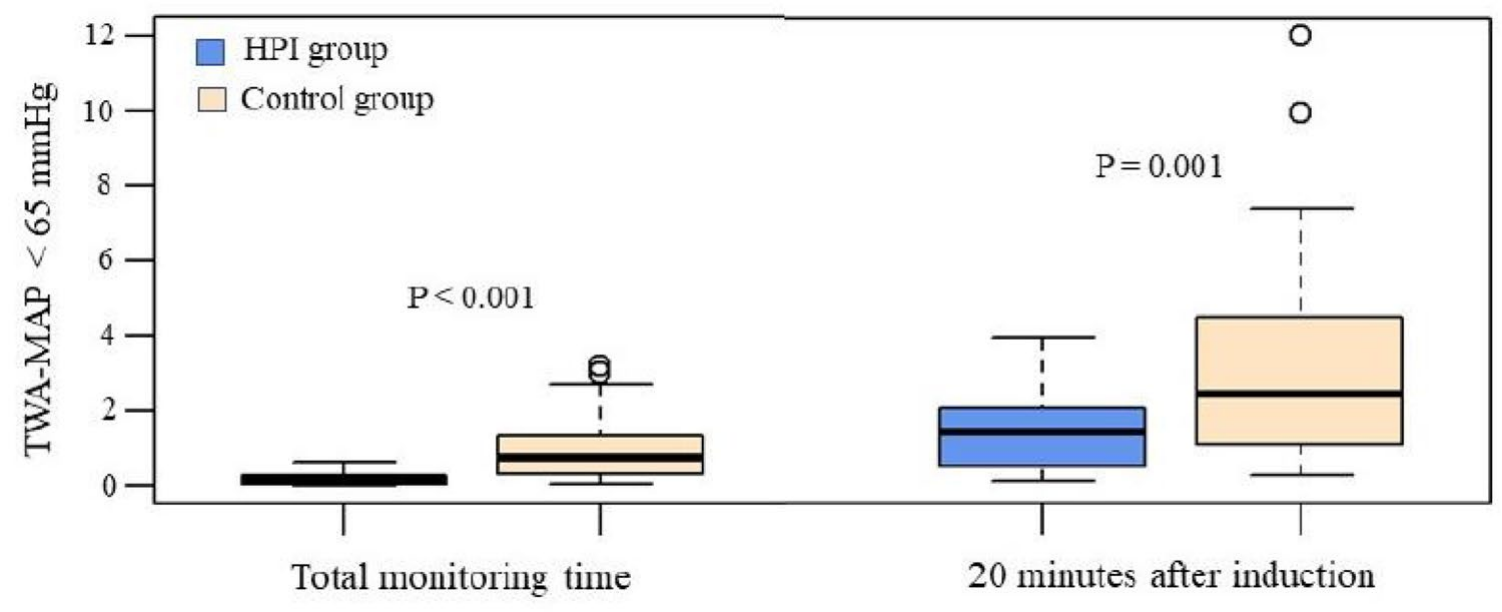
Distribution of TWA-MAP for the overall duration of surgery at the threshold of 65 mmHg by randomized monitoring type. MAP: Mean arterial pressure. TWA: time weighted average. TWA-MAP is expressed in mmHg

The median TWA-MAP < 65 mmHg was 0.14 mmHg (0.04 to 0.66 mmHg) in the HPI group and 0.77 mmHg (0.36 to 1.30 mmHg) in the Control group, with a Hodges-Lehman estimator of -0.57 mmHg (95% CI: − 0.95 to − 0.32, *P* < 0.001), as shown in Table 2 and Fig. 3.

**Table 2:** Comparison of Randomized Groups on cumulative hypotension and severe hypotension

Total monitoring time				
	HPI group (N = 30)	Control group (N = 30)	Hodges-Lehman estimation* (95% CI)	*P* value§
Primary Outcome				
TWA-MAP < 65 mmHg per patient, mmHg	0.14 (0.04, 0.66)	0.77 (0.36, 1.30)	− 0.57 (− 0.95 to − 0.32)	< 0.001
Monitoring time, min	253 (201, 324)	325 (220, 387)	− 43 (− 110 to 11)	0.147
Secondary Outcomes				
Hypotension (MAP < 65 mmHg)				
Hypotensive events in dataset	97	313		
Patients with hypotensive events	30 (100)	30 (100)		> 0.999†
Hypotensive events per patient	2 (1, 5)	7 (5, 13)	− 5 (− 8 to − 3)	< 0.001
Duration of hypotensive events per patient, min	7 (2, 12)	35 (20, 81)	− 29 (− 56 to − 18)	< 0.001
Percentage of time with MAP < 65 mmHg	2.7 (0.9, 4.2)	13.7 (6.9, 24.4)	− 10 (− 18.4 to -5.9)	< 0.001
Area for MAP < 65 mmHg per patient, mmHg·min	46 (10, 73.5)	221.3 (141, 427.4)	− 170.1 (− 272.7 to − 108.3)	< 0.001
Severe Hypotension (MAP < 50 mmHg)				
Severe hypotensive events in dataset	5	14		
Patients with severe hypotensive events	5 (17)	10 (33)		0.233†
Hypotensive events per patient	1 (1, 1)	1 (1, 2)	− 0.00 (− 1 to 0)	0.129
Duration of hypotensive events per patient, min	2 (2, 2)	4 (2, 7)	− 1 (− 6 to 0.3)	0.134
Percentage of time with MAP < 50 mmHg	0.74 (0.48, 0.96)	1.02 (0.64, 1.96)	− 0.38 (− 5.81 to 0.29)	0.254
Area for MAP < 50 mmHg per patient, mmHgmin	8.6 (4.6, 23.3)	19.5 (8.3, 32.6)	− 6.5 (− 31.3 to 12)	0.371
TWA-MAP < 50 mmHg per patient, mmHg	0.03 (0.02, 0.07)	0.04 (0.02, 0.13)	− 0.01 (− 0.29 to 0.05)	0.594
Hypertension (MAP > 110 mmHg)				
Hypertensive events in dataset	21	4		
Patients with hypertensive events	15 (50)	4 (14)		0.005
Hypertensive events per patient	0.5 (0, 1)	0 (0, 0)	0 (0–0)	0.003
Duration of hypertensive events per patient, min	0.7 (0, 3.7)	0 (0, 0)	0 (0–1.66)	0.004
Percentage of time with MAP > 110 mmHg	3.3 (0, 18.3)	0 (0, 0)	0 (0–8.33)	0.004
Area for MAP > 110 mmHg, mmHg·min	4.7 (0, 39.7)	0 (0, 0.3)	1.67 (0–1.17)	< 0.001
TWA-MAP > 110 mmHg per patient, mmHg	0.23 (0, 1.98)	0 (0, 0.17)	0.08 (0–0)	< 0.001
Severe Hypertension (MAP > 130 mmHg)				
Severe hypertensive events in dataset	5	1		
Patients with severe hypertensive events	4 (14)	1 (3)		0.353†
Severe hypertensive events per patient	0 (0, 0)	0 (0, 0)	0 (− 0 to 0)	0.189
Duration of severe hypertensive events per patient, min	0 (0, 0)	0 (0, 0)	0 (− 0 to 0)	0.206
Percentage of time with MAP > 130 mmHg	0 (0, 0)	0 (0, 0)	0 (− 0 to 0)	0.206
Area for MAP > 130 mmHg (AUT), mmHg·min	0 (0, 3)	0 (0, 0)	0 (− 0 to 0)	0.011
TWA-MAP > 130 mmHg per patient, mmHg	0 (0, 0.15)	0 (0, 0)	0 (− 0 to 0)	0.011

Data are presented as N (%) or median (IQR)

*HPI* Hypotension Prediction Index, *TWA* Time-weighted average. *MAP* mean arterial pressure

^*^Hodges-Lehman estimation of location shift and 95% CI. §*P* value corresponded to Wilcoxon rank sum test. †*P* value corresponded to Chi Square test or Fisher’s exact test in case of expected frequencies < 5

All patients in both groups experienced hypotensive events with MAP < 65 mmHg. However, the number of hypotensive events was lower in the HPI group: 97 in the HPI group vs 313 in the Control group. Median number of hypotensive events per patient were 2 (1–5) in the HPI group and 7 (5–13) in the Control group. Median duration of hypotensive events per patient was 7 min (2–12 min) in the HPI group and 35 min (20–81 min) in the Control group, corresponding to a 2.7% (0.9–4.2%) and 13.7% (6.9–24.4%) of total monitoring time in the HPI group and in the Control group, respectively (*P* < 0.001). The AUC for MAP < 65 mmHg per patient was 46 mmHg·min (10–73.5 mmHg·min) in the HPI group and 221.3 mmHg·min (141–427.4 mmHg·min) in the Control group, with a Hodges-Lehman estimator of − 170.1 mmHg·min (95% CI, − 272.7 to − 108.3 mmHg·min, *P* < 0.001).

The TWA for MAP < 50 mmHg was 0.03 mmHg (0.02–0.07 mmHg) in the HPI group and 0.04 mmHg (0.02–0.13 mmHg) in the Control group, with a Hodges-Lehman estimator of − 0.01 mmHg (95% CI − 0.29 to 0.05 mmHg, *P* = 0.594). In the HPI group, 5 patients (17%) compared to 10 (33%) patients in the Control group experienced severe hypotension with MAP < 50 mmHg. Total number of severe hypotensive events was 5 in the HPI group and 14 in the Control group. Median number of severe hypotensive events was 1 (1–1) in the HPI group and 1 (1–2) in the Control group (*P* = 0.129). Median duration of severe hypotensive events was 2 min (2–2 min) in the HPI group compared to 4 min (2–7 min) in the Control group, corresponding to 0.74% (0.48–0.96%) of total monitoring time in the HPI group and 1.02% (0.64–1.96%) of total monitoring time in the Control group (*P* = 0.254), as shown in Table 2. During the first 20 min after induction of GA, the median TWA-MAP < 65 mmHg was 0.53 mmHg (0.06, 1.8 mmHg) in the HPI group and 2.15 mmHg (0.65, 4.2 mmHg) in the Control group, with a Hodges-Lehman estimator of − 1.29 mmHg (95% CI, − 2.51 to − 0.36 mmHg,* P* = 0.001), as displayed in Table 3 and Fig. 3.

**Table 3:** Comparison of Randomized Groups on hypotension and severe hypotension during first 20 min after induction of general anesthesia (GA)

First 20 min after GA induction	HPI group	Control group	Location shift* (95% CI)	*P* value§
Hypotension (MAP < 65 mmHg)				
Hypotensive events in dataset	26	49		
Patients with hypotensive events	20 (67)	28 (94)		0.021†
Hypotensive events per patient	1 (1, 1)	2 (1, 2)	− 1 (− 0 to 0)	0.008
Duration of each hypotensive event, min	3.6 (2.3, 6.08)	8 (5,10)	− 4 (− 6.3 to − 1.3)	0.002
Percentage of time with MAP < 65 mmHg	18 (12, 30)	39 (27, 53)	− 20 (− 31 to − 6.6)	0.002
Area for MAP < 65 mmHg (AUT) per patient, mmHg × min	29 (10.6, 40.5)	49 (22.7, 37)	− 22.3 (− 47 to − 3)	0.025
Time-weighted average of area for MAP < 65 mmHg per patient, mmHg	0.53 (0.06, 1.8)	2.15 (0.65, 4.2)	− 1.29 (− 2.51 to − 0.36)	0.001
Severe Hypotension (MAP < 50 mmHg)				
Hypotensive events in dataset	1	7		
Patients with hypotensive events	1 (3)	5 (17)		0.195†
Hypotensive events per patient	1	1 (1–2)		0.070
Duration of hypotensive events per patient, min	1.7	4.3 (1.3–6.6)		0.072
Percentage of time with MAP < 50 mmHg	8	21 (6, 33)		0.072
Area for MAP < 50 mmHg per patient, mmHg × min	23.3	30 (4.6, 40)		0.202
Time-weighted average of area for MAP < 50 mmHg per patient	1.16	1.51 (0.23–2)		0.202

Data are presented as median (IQR)

*HPI* Hypotension Prediction Index group, *MAP* mean arterial pressure

^*^Hodges-Lehman estimation of location shift and 95% CI. §* P* value corresponded to Chi Square test or Fisher’s exact test in case of expected frequencies < 5

Hypertensive events (MAP > 110 mmHg) were uncommon, but more frequent in the HPI group than in the Control group (Table 2). Total number of hypertensive events with MAP > 110 mmHg for more than 1 min were 21 in the HPI group compared to 4 in the Control group. The median incidence of hypertension was 0.5 (0–1) hypertensive episodes per patient in the HPI group vs 0 (0–0) in the Control group, with a Hodges-Lehman estimator of 0 (95% CI: 0–0) episodes per patient (*P* = 0.003). Median duration of hypertension was 0.7 min (0–3.7 min) per patient in the HPI group and 0 min (0–0 min) in the Control group. The median TWA-MAP > 110 mmHg was 0.23 mmHg (0–1.98 mmHg) in the HPI group vs. 0 mmHg (0–0.17 mmHg) in Control group, with a Hodges-Lehman estimator of 0.08 (95%CI: 0–0 mmHg, *P* < 0.001). No difference was detected between the two groups regarding the incidence of severe hypertension (MAP > 130 mmHg), as shown in Table 2.

Comparing intraoperative therapeutic management, HPI patients received more dobutamine [15 (50%) patients vs 0] and cumulative dose of noradrenaline [962 mcg (419– 2249 mcg) vs 539 mcg (385 vs 703 mcg), *P* = 0.041] (Table 4). There were no other differences in the cumulative dose of other vasopressors, fluids, or blood transfusion.

**Table 4:** Comparison of Randomized Groups on Cumulative Doses of Reported Medications Given During Surgery

	HPI group	N (%)	Control group	N (%)	Location shift* (95% CI)	*P* value§
Noradrenaline, mcg	962 (419, 2249)	29 (97)	539 (384, 703)	17 (57)	423 (3–1155)	0.041
Ephedrine, mg	10 (5, 10)	3 (10)	10 (5, 10)	7 (23)	− 0 (− 5 to 5)	0.893
Etilefrine, mg	2 (2, 2)	2 (7)	4 (2, 7)	17 (57)	− 2 (− 10 to 1)	0.306
Fluids, ml	2250 (2000, 4000)	30 (100)	2150 (1500, 3375)	30 (100)	250 (− 500 to 1000)	0.402
Colloids, ml	500 (500, 1000)	14 (47)	500 (500, 1000)	16 (53)	0 (− 0 to 0)	0.617
Concentrated red blood cells, ml	500 (250, 750)	5 (17)	250 (250, 500)	5 (17)	− 250 (− 999 to 250)	0.366
Dobutamine, mcg	7607 (2231, 15439)	15 (50)	–	0 (0)		
Propofol, mg	120 (110, 140)	30 (100)	120 (110, 139)	30 (100)	0 (− 10 to 10)	0.486
Sufentanil (intravenous), mcg	35 (30, 40)	30 (100)	35 (30, 45)	30 (100)	− 5 (− 5 to 0)	0.198

Data are presented as median (IQR)

*HPI* Hypotension Prediction Index group, *MAP*: mean arterial pressure

^*^Hodges-Lehman estimation of location shift and 95% CI. §*P* value corresponded to Wilcoxon rank sum test

Total reported treatments were 358 in the HPI group and 187 in the Control group; median number of treatments was 9 (4–16) in the HPI group vs 6 (4–10) in the Control group, with a Hodges-Lehman estimator of 2 treatments (95% CI: 0–7, *P* = 0.078).

The median time from the HPI alert of 85 to the first treatment was 60 s (20–140 s) in the HPI group, and from hypotension to the treatment in the Control group 190 s (78–431 s), with a Hodges-Lehman estimator of − 119 s (95% CI: − 161 to − 72 s, *P* < 0.001).

Advanced hemodynamic variables (CO, CI and SV) in the 2 groups are showed in Table 5.

**Table 5:** Comparison of Randomized Groups on advanced hemodynamic variables during total monitoring time

Total monitoring time				
	HPI group (n = 30)	Control group (n = 30)	Median difference* (95% CI)	*P* value§
CO, L/min	4.0 (3.4, 4.9)	3.9 (2.9, 4.8)	0.29 (0.20–0.29)	< 0.001
CI, L/min/m^2^	2.8 (2.1, 3.3)	2.4 (2.0, 3.0)	0.29 (0.20–0.29)	< 0.001
SV, mL/beat	60 (50,72)	56 (48, 68)	2.00 (2.00–2.99)	< 0.001

Data are presented as median (IQR)

*HPI* Hypotension Prediction Index group, *CO* cardiac output, *CI* cardiac index, *SV* stroke volume

^*^Median difference and 95% CI estimated using Hodges-Lehmann estimator. § *P* value corresponded to Wilcoxon rank sum test

In the HPI group, pleural effusion occurred in 1 (3%) patient within the first postoperative week vs 6 patients (20%) in the Control group. Cardiac arrythmias were reported in 1 (3%) patient in the HPI group and in 2 (6%) patients in the Control group. In the Control group one patient (3%) reported cardiac ischemia and one patient (3%) died within a month of surgery.

## Discussion

This study demonstrated that application of a machine learning–derived predictive algorithm in combination with a pre-defined GDT hemodynamic protocol reduced IOH as measured by TWA-MAP in patients undergoing major gynaecologic oncologic surgery compared to a standard GDT-based hemodynamic optimization. Also, hypotension after GA induction were significantly reduced by adopting an HPI-based “pro-active” hemodynamic protocol, in which the physician intervenes before the adverse event has occurred.

Several studies investigated the role of GDT during non-cardiac surgery in reducing postoperative complications and mortality, with mixed results [16, 17, 32, 33]. On the other hand, intraoperative fluid optimization may not be sufficient to reduce the risk of IOH [10, 18, 20]. Patients enrolled in these studies received GDT fluid therapy as standard practice, despite which the incidence of IOH remained significant. This would suggest that fluid optimization alone is not a sufficient therapy to prevent IOH for patients undergoing major surgery. Intraoperative maintenance of adequate organ perfusion is likely to reduce postoperative complications, and GDT uses defined goals of care aimed to optimize organ perfusion. On the other hand, vasoactive drugs used in the perioperative period to support organ perfusion may reduce postoperative complications and hospital length of stay in adult patients undergoing major abdominal surgery [18, 34].

As excellently pointed out in a recent paper by Saugel et al., the most common haemodynamic target variables are blood flow variables (CO), dynamic cardiac preload variables (PPV or SVV), and arterial pressure, but targeted arterial pressure management, surprisingly, is not properly considered GDT [35]. Haemodynamic treatment strategies need specific haemodynamic target variables and values that trigger specific interventions (vasopressor, fluids, inotrope or a combination of these): all of these haemodynamic treatment strategies can be counted as “GDT”, but they are used in very different ways with different effects on outcome [10, 19, 32–35]. In this study we tried to define a specifically “modified” GDT protocol that took into account blood pressure as well as flow. In fact, even if HPI accurately predicts the occurrence of arterial hypotension, clinicians need to intervene quickly to prevent it. Moreover, administering adequate therapy is also a challenge, since it requires a proper interpretation of the hemodynamic parameters to elucidate the underlying mechanisms leading to increased hemodynamic instability. In previous studies, different authors have tried to interpret advanced hemodynamic parameters including SVV, Ea_dyn_ and dP/dt_max_ to determine the pathophysiological mechanisms leading to hypotension [24, 25, 36]. We tried to define a simpler algorithm to allow the clinician a prompt therapeutic choice. It is not surprising that the median time from alarm (in the HPI group) or hypotension (Control group) to treatment was three times higher in the Control group.

The fluid balance in the two groups was not significantly different. Conversely, in the HPI group, a higher cumulative dose of noradrenaline was observed. In this regard, our results differ from those by Wijnberge et al. [25]. In our study, the amount of IOH in the Control group as determined by TWA-MAP is significantly higher than reported by Maheshwari and by Wijnberge, and similar to Tsoumpa [24, 25, 36]. In a recent large RCT Maheshwari et al. stated that HPI guidance failed in reducing IOH during noncardiac surgery [24]. On the other hand, the authors observed half the hypotension expected in the Control group, probably due to an aggressive hypotension reduction strategy (*e.g.*, Hawthorne effect) [24].

One possible explanation for our results could be the use of combined neuraxial-general anesthesia in our patient cohort. For major gynaecologic oncologic surgery, a multimodal, opioid sparing analgesic strategy with neuraxial analgesia is recommended [37]. However, the use of epidural analgesia is reported to significantly increase the incidence of IOH and the need for vasopressor [38, 39]. Furthermore, other risk factors for IOH were female sex and major demolitive surgery of our cohort [29, 40].

It is worth noting that in HPI group 50% of patients received dobutamine. The small number of the sample does not allow us to analyze the role of a single drug on our results; however, a non-invasive monitoring of cardiac contractility (dP/dt_max_) adds this therapeutic weapon to the clinician in a non-cardiac surgery setting.

It seems likely that during surgery, in certain situations, the need for vasopressors indicates depression of the cardiovascular system due to excessive depth of anesthesia. Monitoring depth of anesthesia could be also useful to personalize anesthetic dosage and to reduce vasopressor support [39, 41]. We carefully monitored depth of anesthesia with BIS in the two groups, but this did not eliminate the need for vasopressor support.

Another important finding in our study is the amount of hypotension after induction. Our analysis highlights that 67% of patients in HPI group and 94% in Control group developed hypotension after GA induction. These values are higher than those reported in literature [15, 20, 42]. Khan et al. showed that in a predominantly ASA 3 (or higher) surgical population, fluid optimization of cardiac preload did not reduce the degree of haemodynamic impact from GA induction [20]. In a recent study in non-cardiac surgery patients, Saugel et al. reported that GA induction was associated with a significant reduction in arterial pressure and systemic vascular resistance [42]. Our data show that hypotension following induction of GA was significantly reduced in the HPI group, suggesting that early intervention may be effective to prevent hypotension from the beginning of the anesthesia. Nevertheless, the role of the hypotensive load during induction and its associated damage is uncertain due to prolonged duration, as well as stressful and hemorrhagic nature of oncologic demolitive surgery.

We may argue that a simple physiologically based algorithm alone (without reliance on HPI), considering GDT and arterial pressure, could result in an important reduction in hypotension exposure. However, it seems reasonable that prediction of impending hypotension would allow the clinician to act with pharmacologic and fluidic interventions early.

Mixed results concerning the risk of overtreatment are reported [23–25, 36]. We chose to carry out the analysis of TWA-MAP for hypertension with predefined thresholds of 110 and 130 mmHg [43, 44]. We found a slightly significant difference between the two groups for the incidence and amount of hypertension (MAP > 110 mmHg), which was higher in HPI group. No differences were detected for severe hypertension (MAP > 130 mmHg). Hypertension is not as strongly associated as hypotension with increased complications and morbidity [2].

This study has several limitations. First, this is a single-centre RCT aimed to test the performance of the HPI algorithm in preventing IOH in a specific surgical and anesthetic setting as major gynaecologic oncologic surgery. Consequently, as previous trials, it was underpowered to investigate differences in clinical outcomes between both hemodynamic managements. Second, we have used a fixed definition of arterial hypotension based on the HPI algorithm. This threshold could vary and should be individualized based on individual patient characteristics and organ perfusion needs. However, this threshold has been frequently used to define IOH and it has been consistently associated with myocardial injury, acute kidney injury, and mortality in noncardiac surgery [9, 10, 12]. Third, the performance of HPI algorithm was not specifically validated during GA induction, though in a recent paper we demonstrated an accurate prediction of hypotension 5 min, 3 min and 1 min before the event, suggesting a high efficacy even in the very early stages of anesthetic procedures [45]. Fourth, the physicians involved in this study were skilled in the use of HPI algorithm and interpretation of the secondary screen variables. So, for an optimal implementation of our simplified HPI-based protocol and to extrapolate it to other settings, a prior training is highly recommended. Fifth, it might seem strange that in Control group the inotrope use was not planned, despite being a cardiac output-guided management. As mentioned before, our intention was to compare an HPI-guided hemodynamic protocol with our routine institutional management, which does not involve the use of an inotrope for non-cardiac surgery, and this could represent a bias given the frequent incidence of episodes of decreased cardiac contractility during major abdominal surgery [46].

In conclusion, this study demonstrated that application of a machine learning–derived predictive algorithm in combination with a pre-defined GDT hemodynamic protocol reduced IOH and hypotension following induction of anesthesia in patients undergoing major gynaecologic oncologic surgery compared to a standard GDT management. Further research is needed to determine the impact of this pro-active hemodynamic treatment on clinical outcomes.

## Data Availability

The datasets used and/or analysed during the current study are available from the corresponding author on reasonable request.
